# Chemical, Nutritional, Antinutritional, Physical and Technological Characterization of Breads Containing Germinated and Non-Germinated Black Lentil Flours Under Different Fermentation Conditions

**DOI:** 10.3390/molecules31040619

**Published:** 2026-02-10

**Authors:** Christine (Neagu) Dragomir, Sylvestre Dossa, Ariana Velciov, Daniela Stoin, Ileana Cocan, Florina Radu, Călin Jianu, Ersilia Alexa

**Affiliations:** 1 Faculty of Food Engineering, University of Life Sciences “King Mihai I” from Timisoara, Aradului Street No. 119, 300645 Timisoara, Romania; christine.neagu@usvt.ro (C.D.); dossasylvestre@usvt.ro (S.D.); danielastoin@usvt.com (D.S.); ileanacocan@usvt.ro (I.C.); florinaradu@usvt.ro (F.R.); calinjianu@usvt.ro (C.J.); 2 “Food Science” Research Center, University of Life Sciences “King Mihai I” from Timisoara, Aradului Street No. 119, 300645 Timisoara, Romania; 3 Doctoral School, University of Life Sciences “King Mihai I” from Timisoara, Aradului Street No. 119, 300645 Timisoara, Romania

**Keywords:** yeast, sourdough, total polyphenols, phytic acid, color parameters

## Abstract

This study aims to investigate the possibility of using lentil flour in its native and germinated form as microgreen in bread-making technology, as well as how the fermentation process (with yeast or sourdough) influences the chemical, nutritional, antinutritional, physical and technological parameters of the bread. For this purpose, 14 bread samples were obtained using composite flours (wheat flour and black lentil flour) with the addition of 10, 20, and 30% lentil flour (LF) relative to wheat flour (WF), as well as composite flours (wheat flour and germinated lentil powder GL) in proportions of 2.5, 5, and 7%. Each flour sample was used in bread production using the direct fermentation method with yeast (yeast lentil bread BLY and yeast germinated lentil bread BGLY) and the indirect method with sourdough (sourdough lentil seed bread BLS and sourdough germinated lentil bread BGLS). Experimental results regarding nutritional composition showed a significant increase in protein content compared to the control (wheat flour bread), with the highest value recorded in the sample with 7.5% germinated lentil fermented with sourdough (29.18%), which also stood out for the highest total polyphenol content (1183.84 mg/100 g) and the lowest phytic acid content. Regarding the physical properties of the bread, an increase in elasticity, porosity, and height/diameter ratio was observed in the samples with an intermediate addition of lentil flour (20%) and germinated lentil flour (7.5%). The physical color parameters of the final product are also significantly influenced by the addition of black lentil flour, as well as germinated lentils. In conclusion, it can be stated that the use of lentil flour in its germinated form increases the nutritional and functional properties of bread, while the use of sourdough in the technological process leads to a decrease in the phytic acid content of the samples. Among the tested formulations, the addition of 20% lentil flour or 5% lentil germinated lentil resulted in the most favorable balance between bread elasticity, porosity, and H/D ratio.

## 1. Introduction

Bread remains one of the most consumed foods globally, but products made predominantly from refined wheat flour often have a low density of bioactive compounds and may contain antinutritional factors that limit mineral bioavailability. In this context, the development of bread varieties with improved nutritional and functional value, through the use of sustainable raw materials rich in protein, fiber and phytonutrients, is a current direction in bakery research.

Legumes, including lentils (*Lens culinaris*), are considered promising ingredients for the fortification of bakery products due to their high protein, resistant starch, dietary fiber and polyphenol content, as well as their favorable micronutrient profile [1].

Among the varieties, black lentils stand out for their content of phenolic compounds and pigments with antioxidant potential, but their use in baked goods is limited by the presence of antinutritional factors (especially phytic acid) and by the impact on technological and sensory properties (color, texture, volume). Phytic acid can chelate minerals such as iron, zinc or magnesium, reducing their absorption, which is why its reduction by bioprocessing is a frequent objective in the formulation of functional foods [2]. The addition of legume flours modifies the color parameters of the crumb and crust, with a typical increase in the a* (reddish shades) and b* (yellowish shades) values, which can influence consumer acceptability and must be correlated with the level of substitution and fermentation technology [3].

Sprouting is a relatively mild bioprocessing technology, capable of activating endogenous enzymes (e.g., phytases), initiating metabolic transformations, and leading to a decrease in antinutrients, while increasing or modifying phenolic fractions and antioxidant capacity. The recent literature generally indicates trends of phytic acid reduction during germination and, in many cases, increases in polyphenol content, although the magnitude of the effects depends on the variety, germination conditions, and matrix [2,4]. For black lentil-based ingredients, improvements in the bioactive profile and decreases in phytate have been explicitly reported in flours/compounds obtained from germinated seeds, suggesting a high potential for composite flour applications in bakery [5].

In addition to germination, dough fermentation is a critical step that can amplify the nutritional and technological effects. Yeast fermentation (*Saccharomyces cerevisiae*) is the most widely used industrially to obtain the volume and structure of the crumb, but sourdough fermentation, based on a consortia of yeasts and lactic acid bacteria, generates a more complex acidification and a broader spectrum of metabolites (organic acids, enzymes, exopolysaccharides) with an impact on digestibility, aromas and nutrient bioavailability [6,7].

The specific acidification of sourdough favors phytate degradation both by activating native flour phytases and by microbial enzymatic contribution, and recent experimental evidence and syntheses support the ability of sourdough fermentation to significantly reduce phytate and improve mineral accessibility [7,8,9].

The integration of legume flours into bread, including yeast-fermented variants, has been associated with increases in phenolic compound content and antioxidant activity, but the effects are dependent on the type of legume, the level of substitution, and the process parameters (hydration, fermentation time/temperature) [3,7].

In this context, the systematic comparison of germinated versus non-germinated black lentil flour, under different fermentation regimes (yeast vs. sourdough), is relevant both for clarifying the mechanisms (phenolic and phytate transformations) and for optimizing the quality of the finished product (color, composition, functional potential).

Therefore, the aim of this paper was the nutritional and technological characterization of breads formulated with the addition of germinated and non-germinated black lentil flour, obtained under distinct fermentation conditions (with yeast and with sourdough, respectively). Specifically, (i) the nutritional value of the formulated products, (ii) the phytic acid content as an indicator of potential mineral bioavailability, (iii) the total polyphenol content as a marker of antioxidant potential and (iv) the color parameters (L*, a*, b*) of the bread were evaluated, in order to correlate the biochemical changes induced by germination and fermentation with the technological and perceptible attributes of the finished product. The results are useful for the design of bakery products with an improved profile of bioactive compounds, while maintaining the characteristics of the product.

## 2. Results and Discussion

### 2.1. Physical Characteristics of Bread Samples

The physical characteristics (elasticity, porosity and height-to-diameter (H/D) ratio) of bread samples are shown in Table 1. An evaluation of the physical characteristics of the bread formulations revealed statistically significant differences (*p* < 0.05) between the control samples C1 (bread leavened with yeast) and C2 (bread leavened with sourdough) and the experimental variants in which wheat flour (WF) was partially replaced with 10–30% lentil flour (LF) and 2.5–7.5% germinated lentil flour (GLF). The experimental design enabled the assessment of the impact of WF substitution with LF and GLF on the primary physical parameters, including porosity, elasticity, and H/D ratio, in comparison to the control samples. The study analyzed two sets of samples: Group I consists of yeast-leavened bread, including the control sample C1, samples enriched with LF (BLY1, BLY2, BLY3) and those enriched with GLF (BGLY1, BGLY2, BGLY3). Group II consists of bread leavened with sourdough, with control sample C2 and the same formulation variants (BLY1, BLY2, BLY3, and BGLY1, BGLY2, BGLY3).

The control sample (C1) exhibited the lowest values of elasticity, porosity and H/D ratio in contrast to the samples enriched with LF and GLF, which consistently demonstrated improvements across these physical attributes. The elasticity showed a moderate increasing trend with the incorporation of LF, rising from 91.857% in BLY1 to 92.267% in BLY2, followed by a decrease to 91.440% in BLY3. A comparable pattern was observed in the GLF formulations, where the elasticity increased from 93.073% in BGLY1 to 94.153% in BGLY2 before declining to 91.625% in BGLY3. Similar results were also reported in another study, which highlighted that amylolytic enzymes improve the physical properties of bread and the viscoelastic behavior of wheat dough. The porosity likewise exhibited an upward trend with increasing substitution levels of WF by LF and GLF. In the LF-enriched samples, the porosity increased from 67.790% (BLY1) to 68.100% (BLY2) and then decreased to 66.177% in BLY3, with all values remaining above the control. In the GLF formulations, the porosity rose from 68.340% (BGLY1) to 69.680% (BGLY2) and subsequently declined to 66.610% in BGLY3, yet still exceeded that of the control sample (66.087%). The decrease in elasticity/porosity at higher addition levels can be explained by the nature of the microgreens: powders from vegetative tissue are relatively rich in fibers and structural components that dilute gluten and increase water absorption, affecting gas retention and crumb structure. Similarly, the literature on breads enriched with microgreens/green tissue reports the need for moderate addition levels to avoid deterioration of volume and texture parameters [10].

A similar behavior was observed for the H/D ratio. Notably, all H/D values in the enriched formulations surpassed the control value, reinforcing the positive effect of LF and GLF incorporation on the product structure. Similar studies have shown that the controlled germination of durum wheat grains can improve bread quality, particularly its volume and porosity [11,12].

In the subsequent stage of the evaluation, the samples from Group II namely, the breads leavened with sourdough, were examined to determine the extent to which the trends previously observed in yeast-leavened samples were maintained under this fermentation system. The control sample (C2) exhibited lower values of elasticity (92.140%), porosity (67.607%), and H/D ratio (0.546) compared with the LF- and GLF-enriched sourdough samples; however, these values remained higher than those recorded for the yeast-leavened control (C1). The behavior observed in Group II reflected the same overall variation pattern identified in Group I, although the values of elasticity, porosity, and H/D ratio were consistently higher. This indicates a favorable influence of sourdough fermentation on the physical performance of breads. The highest values of the physical parameters in Set II were recorded for the BLS2 and BGLS2 formulations. Likewise, the porosity increased substantially, reaching 72.607% in BGLS2, compared with 67.607% in C2. The H/D ratio also improved markedly, attaining 0.590 in BGLS2, relative to 0.546 in the control sample C2. Overall, these findings highlight clear differences in the structural behavior of breads depending on the fermentation type. Sourdough-fermented samples consistently exhibited higher elasticity, porosity, and H/D ratios compared with both their respective controls and the equivalent yeast-leavened variants, underscoring the superior contribution of sourdough fermentation to structural quality. These enhancements indicate the ability of germinated malt to promote the formation of a more coherent protein–starch network, capable of retaining fermentation gases more efficiently. In both fermentation systems, the addition of lentil flour (LF) and germinated lentil flour (GLF) led to notable improvements in physical parameters; however, the effects were more pronounced in the samples fermented with sourdough. Moreover, the samples in Group II exhibited higher values for these parameters than those in Group I, suggesting a synergistic interaction between GLF incorporation and sourdough fermentation. The improvement in physical properties in GLF formulations can be attributed to the favorable modifications induced by germination, including increased protein solubility [13], enhanced proteolytic activity, and altered starch behavior—changes that are further amplified by lactic–acetic fermentation. The interplay between these processes contributes to a more aerated, elastic, and structurally stable crumb. Overall, the findings indicate that sourdough fermentation combined with partial substitution of wheat flour with GLF represents the most effective strategy for optimizing the physical quality of rolls, outperforming traditional yeast fermentation. These observations are consistent with previously published studies, which have identified germination as a viable method for improving the physical characteristics of bread. Earlier research has demonstrated that germination positively influences the behavior of flour during breadmaking [11,12,14,15].

### 2.2. Nutritional Composition of Bread Samples

The nutritional composition of the bread samples, expressed as the moisture, protein, fat, carbohydrates and energy value, is presented in Table 2. The samples analyzed were the same as those presented in Section 2.1.

The nutritional characteristics of the bread samples were significantly influenced by both the type of added ingredient (lentil seeds vs. germinated lentil as microgreens) and the fermentation method (yeast vs. sourdough).

The yeast-fermented samples generally showed lower moisture values compared to sourdough-fermented samples. The increase in moisture in sourdough breads (BLS and BGLS) can be attributed to the higher water retention capacity associated with both lactic acid fermentation and the increased protein and fiber content from lentils, an effect frequently reported for sourdough [16,17].

The ash content increased progressively with the level of lentil substitution, reaching maximum values in samples with 30% lentil seeds (BLY3: 2.23%; BGS3: 2.67%). This trend reflects the superior mineral content of legumes compared to refined wheat flour and confirms the potential of composite breads as improved sources of micronutrients [18].

A clear dose-dependent effect was observed for the protein content. Bread fortified with lentil seed flour (BLY and BLS) and especially germinated lentil (BSLY and BGLS) showed significant increases in protein compared to the control samples (C1 and C2). The highest values were observed in the sourdough-fermented samples (BGLS3: 29.18%), highlighting both the high nutritional value of the germinated lentil and the favorable effect of sourdough fermentation on protein retention [19].

The lipid content was relatively low in all samples (<4%), but a slight increase was observed in the sourdough-fermented lentil seed breads (BLS1–BLS3). In contrast, the BGLS formulas showed lower lipid values, possibly due to the mobilization and degradation of lipid fractions during germination [20].

The carbohydrate content progressively decreased in bread fortified with germinated powder, especially in those fermented with sourdough (BGLS), where values dropped to 30.37 g/100 g (BGLS3). This reduction is correlated with the dilution of wheat starch and the utilization of fermentable carbohydrates by sourdough-specific microbiota [16,21].

The energy value was the result of the balance between the increase in proteins and the reduction in carbohydrates and fats. The yeast and lentil seed samples (BLY) showed the highest energy values (up to 288.21 kcal/100 g), while the sourdough and lentil seed samples (BLS) recorded the lowest values (230.57–254.90 kcal/100 g). This result confirms that sourdough fermentation can lead to products with a lower energy density, despite a high protein content [17,22].

Overall, the data indicate that the addition of germinated lentil as microgreens powder in wheat flour, especially in combination with sourdough fermentation, allows the production of breads with high protein content, superior mineral contribution, and moderate energy density, supporting their potential for developing functional bakery products.

The changes in the proximate composition and mineral fraction observed after the addition of microgreens are supported by the literature [23], which describes microgreens as a plant matrix rich in micronutrients and phytochemicals, with a mineral and phenolic profile influenced by the species and growing conditions.

Sourdough fermentation significantly influenced the nutritional parameters of bread compared to yeast fermentation. In general, the sourdough samples showed higher moisture, associated with an increased water retention capacity, as a result of lactic bacteria activity and structural changes in the dough matrix. Additionally, sourdough fermentation led to lower carbohydrate content and energy values, an effect attributed to the consumption of fermentable carbohydrates and the production of organic acids.

At the same time, sourdough breads exhibited an improved mineral profile, reflected in higher ash contents, as well as potentially increased nutrient bioavailability, due to the activation of endogenous phytases in the more acidic environment of lactic fermentation. Compared to yeast, sourdough favored the production of products with moderate energy density and superior nutritional quality, especially in the formulations enriched with lentils or lentil microgreens.

### 2.3. Antinutritional Compounds of Bread Samples (Phytic Acid)

**Figure 1:**
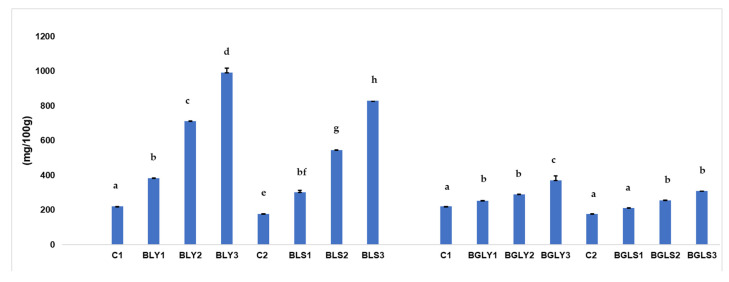
Phytic acid composition of bread obtained with WF fortified with LF and GLF using yeast and sourdough fermentation. The values are expressed as mean ± standard deviation; data sharing different letters in the same group are significantly different (*p* < 0.05).

The phytic acid content of the analyzed samples varied significantly depending on the type of added ingredient (lentils vs. lentil microgreens) and the fermentation method (yeast vs. sourdough), as shown in Figure 1.

The control sample obtained by yeast fermentation showed a value of 220.00 mg/100 g, while sourdough fermentation led to a lower value (177.28 mg/100 g). This difference confirms the higher efficiency of sourdough in reducing phytic acid, even under short fermentation conditions, due to the faster decrease in pH and the activation of endogenous phytases in wheat flour [9].

In breads with the addition of lentils, a progressive and dose-dependent increase in phytic acid was observed. In yeast fermentation, the values increased from 382.56 mg/100 g (10%) to 712.45 mg/100 g (20%) and 990.80 mg/100 g (30%). A similar behavior was observed in the samples fermented with sourdough but with systematically lower values (300.43–828.09 mg/100 g). These results reflect the high phytate content specific to legume seeds, where phytic acid is the main form of phosphorus storage [24]. In addition, the limited duration of fermentation (2 h) was insufficient to compensate for the increased phytate load introduced by the lentil grains.

In the case of breads with lentil microgreens, the increase in phytic acid was much more moderate. The values ranged from 253.21 to 370.77 mg/100 g in yeast samples and from 210.33 to 310.21 mg/100 g in sourdough samples. Compared to lentils, microgreens resulted in significantly lower levels of phytic acid, due to the activation of phytases during germination, which lead to partial hydrolysis of phytate before incorporation into the dough [25].

The lower phytic acid levels observed in breads enriched with lentil microgreens can be explained by the vegetative nature of the plant material used. Phytic acid is known to be predominantly localized in seeds, where it serves as the main phosphorus storage compound. In contrast, microgreens consist mainly of aerial vegetative tissues, in which phytic acid content is substantially reduced.

The recent literature [26] has reported that microgreens of leguminous and non-leguminous species exhibit significantly lower phytic acid contents compared to their corresponding seeds. For example, studies on pea and sunflower microgreens have demonstrated markedly lower phytic acid levels relative to seed values, confirming that the transition from seed storage tissue to vegetative growth is associated with a decrease in this antinutritional factor. This observation supports the trend recorded in the present study and highlights the nutritional advantage of using microgreens as bread enrichment ingredients. The differences between yeast and sourdough were consistent for all samples analyzed, with sourdough resulting in an additional reduction of approximately 15–25% in phytic acid compared to yeast. This effect is attributed to the more acidic environment created by lactic fermentation, which is favorable for phytase activity [9,16].

The lower phytic acid levels observed in the control bread compared to the lentil-enriched samples can be explained by both the intrinsic phytate content of the raw materials and the limited extent of phytate degradation during short fermentation.

Refined wheat flour (type 650), used in the control formulation, contains relatively low amounts of phytic acid, as phytate is mainly localized in the outer layers and germ of the wheat crumb, which are largely removed during milling. As a result, the initial phytate concentration in the control dough is substantially lower than that of doughs containing lentil flour or germinated lentil [9].

In contrast, lentil seeds are recognized as a rich source of phytic acid, where phytate serves as the principal storage form of phosphorus. Consequently, even partial substitution of wheat flour with lentil flour or germinated lentil leads to an increased phytate load in the dough system and, ultimately, higher residual phytic acid levels in the final bread [24].

Overall, the results highlight that the type of ingredient has a more pronounced impact on the final phytic acid content than the fermentation method, especially under short fermentation conditions. However, the use of germinated lentil and sourdough are effective strategies for limiting phytate levels in enriched bread.

### 2.4. Color Parameters of Bread Samples

Table 3 presents the color parameters of the bread samples analyzed using the CIELAB system [26]. The study was designed to compare formulations under identical conditions, and all color measurements were therefore performed on fresh bread samples (24 h after baking). This approach allowed us to isolate the effect of ingredient type and inclusion level on color, without introducing additional variables related to storage conditions, packaging, or moisture migration.

Color is the main factor influencing consumer perception of the quality of a food product, including bakery and pastry products [27]. Color causes can reflect the degradation of thermolabile compounds, such as carotenoids and phenolic compounds, as well as the transformations generated by Maillard reactions and oxidative processes that occur during baking and storage [28].

The L* values ranged between 64.58 (C1) and 49.30 (BGLS3), indicating a progressive darkening of the core color with increasing levels of vegetable addition (lentil seeds or microgreens) and the use of leaven instead of yeast.

The control breads presented the highest L* values, while the samples with BLY3, BLS3 and BGLY3, BGLS3, especially BLS3 and BGLS3, which were fermented with sourdough, recorded the lowest values, suggesting the intensification of browning reactions and the accumulation of colored pigments during fermentation and baking.

All samples recorded positive a* values (0.17–3.16), indicating a chromatic shift towards red–brown shades. The highest a* values were observed in yeast-fermented bread with microgreens, especially at levels of 5–7.5% microgreens, suggesting an intensification of Maillard reactions and melanoidin formation. Yeast breads generally showed more moderate a* values, indicating a more balanced chromatic profile [29].

The b* values were positive for all samples (11.29–27.71), confirming the golden-yellow dominance specific to bakery products. The highest b* values were obtained for germinated breads (5–7.5%), both yeast and sourdough, reflecting the contribution of natural pigments (carotenoids) and thermal browning products.

Chroma followed a similar trend to b*, ranging from 11.30 to 27.75, indicating an increase in color intensity and saturation with the addition of germ. Breads with microgreens showed the highest C* values, suggesting a more intense and visually appealing color compared to control or lentil breads [29].

The h° values ranged between 82.73° and 89.20°, a range characteristic of golden-yellow hues. The slight decreases in h° observed in the germinated samples indicate a shift towards reddish-brown tones, specific to enriched and more intensively thermally processed functional breads [29]

Overall, it is demonstrated that the addition of lentils and, in particular, microgreens, as well as the use of sourdough, significantly influences the color parameters of bread, causing a reduction in brightness and an increase in chromatic intensity. These changes are associated with intensified Maillard reactions, increased enzymatic activity during fermentation and the contribution of natural pigments from plant raw materials [29].

### 2.5. Total Phenols Content (TPC) of Bread Samples

**Figure 2:**
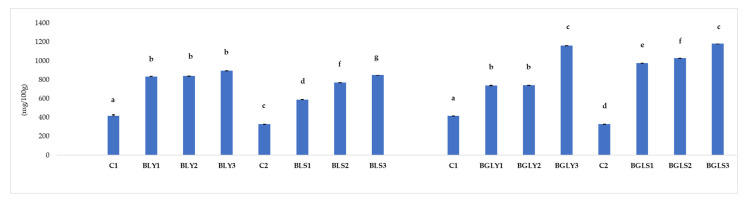
TPC (mg/100 g) of composite yeast and sourdough bread with the addition of lentil seeds and germinated lentil as microgreens. The values are expressed as mean ± standard deviation; data sharing different lettersin the same group (bread with seeds or bread with microgreens) are significantly different (*p* < 0.05).

Bread samples were subjected to total phenol content (mg gallic acid/100 g) calculation, and the obtained values are presented in Figure 2.

Figure 2 illustrates the total phenolic content (TPC) of bread samples formulated with lentil seeds and germinated lentil as microgreens, fermented with either yeast or sourdough. Overall, the results clearly demonstrate that both the type of ingredient (seeds vs. germinated lentil) and the fermentation method (yeast vs. sourdough) significantly influenced the polyphenol content of the final products.

In breads formulated with lentil seeds and yeast fermentation (BLY1–BLY3), the TPC increased progressively with the level of seed addition, from 834.35 mg GAE/100 g (10%) to 894.51 mg GAE/100 g (30%), compared to the control bread (417.21 mg GAE/100 g). This almost two-fold increase confirms that lentil seeds represent an important source of extractable phenolic compounds, which partially resist baking conditions. Similar trends have been reported for breads enriched with legumes, where the inclusion of seeds increases the phenolic reserve and antioxidant potential of the final product [30,31].

When sourdough fermentation was applied to seed-enriched breads (BLS1–BLS3), the TPC values were further improved (590.05–851.40 mg GAE/100 g), indicating that lactic acid fermentation promotes the release of bound phenolic compounds from the cereal–legume matrix. Acidification and microbial enzymatic activity during yeast fermentation are known to hydrolyze phenolic–polysaccharide complexes, thereby increasing the extractability of polyphenols [32,33].

An increase in TPC was observed in breads formulated with germinated lentil, confirming the superior functional potential of germinated raw materials. Breads with yeast-fermented microgreens (BGLY1–BGLY3) showed TPC values ranging from 737.97 to 1161.62 mg GAE/100 g, while breads with sourdough-fermented microgreens (BGLS1–BGLS3) reached the highest levels, up to 1183.84 mg GAE/100 g.

In all formulations, bread with germinated ingredients consistently exhibited higher TPC values than seed-based bread, regardless of the fermentation method. A key and directly relevant study is that of Klopsch et al. (2018) [10], which evaluated bread enriched with microgreens and pea and lupin leaves and tracked changes in secondary metabolites during baking. They showed that, for bread with pea microgreens, a large portion of the flavonoids was found in the final product (relative to the initial material), suggesting that enrichment with microgreens can increase the intake of phenolic compounds in the baked product, although some thermal transformations inevitably occur.

Furthermore, sourdough fermentation systematically improved the TPC compared to yeast fermentation, especially at higher levels of sprout incorporation. These results confirm that combining germination and sourdough fermentation is an effective biotechnological strategy to maximize the polyphenol content of functional bakery products [33].

From a nutritional and functional perspective, the observed TPC values (up to ~1180 mg GAE/100 g) place lentil microgreens-enriched sourdough bread in the upper range reported for polyphenol-rich functional breads, supporting their potential role in antioxidant-enriched diets and health-oriented bakery formulations.

The observed differences in total polyphenol content between sourdough-fermented breads and yeast-fermented breads, depending on the form of lentil addition (microgreens vs. seeds), can be explained by complex interactions between lactic fermentation, the structure of the plant matrix, and the stability of phenolic compounds [8,9].

In sourdough bread with lentil microgreens (BGLS samples), the higher total polyphenol content compared to the corresponding yeast-fermented samples (BGLY) is the result of a synergistic effect between germination and lactic fermentation. Germination activates endogenous enzymes (β-glucosidases, esterases), leading to the release of bound polyphenols and an increase in the extractable phenolic fraction. Sourdough fermentation amplifies this effect through (i) lowering the pH, which favors the solubilization of phenolic compounds; (ii) the enzymatic activity of lactic acid bacteria, capable of hydrolyzing phenolic conjugates; and (iii) reducing polyphenol–protein or polyphenol–fiber interactions, thereby increasing their extractability. In this context, the released polyphenols are relatively stable in the acidic environment of sourdough, and their oxidative degradation is limited, which explains the higher level of total polyphenols in sourdough bread with lentil microgreens compared to yeast-fermented bread [8,9].

In contrast, in sourdough bread with lentil seeds (BLS samples), the total polyphenol content is lower than in yeast-fermented bread (BLY samples). This behavior is explained by the intact and more rigid structure of the seeds, in which a significant proportion of polyphenols is bound to the cell wall or to macromolecules such as proteins and phytate. Under sourdough fermentation conditions, the acidic environment and microbial activity may favor (i) oxidation or degradation of certain sensitive phenolic compounds, particularly simple flavonoids; (ii) the polymerization of phenols, which reduces their detectability by spectrophotometric methods (e.g., Folin–Ciocalteu); and (iii) the formation of phenol–protein/fiber complexes, which are less extractable. In the absence of the germination process, which could have activated mechanisms for the enzymatic release of polyphenols, sourdough fermentation acts primarily as a transforming factor and partially as a degrading one, resulting in lower total polyphenol levels compared to yeast bread with lentil seeds, where the less acidic environment limits these transformations [34,35].

Yeast fermentation, characterized by a more moderate acidification and lower enzymatic activity on phenolic compounds, tends to preserve the existing polyphenols, especially in samples with lentil seeds. Under these conditions, the polyphenols largely remain undecomposed, which explains the higher values compared to sourdough-fermented samples [34].

The differences in total polyphenol content reflect the fact that sourdough does not act uniformly on all forms of lentils. In combination with lentil microgreens, sourdough promotes the release and stabilization of polyphenols, leading to higher values. In contrast, in bread with ungerminated lentil seeds, sourdough fermentation promotes transformations and partial losses of phenolic compounds, resulting in lower values compared to yeast fermentation.

### 2.6. Pearson Correlations

**Figure 3:**
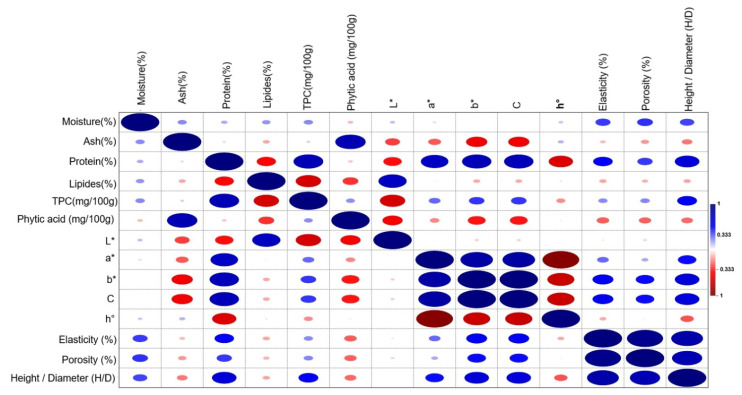
Pearson correlation heatmap of nutritional, antinutritional, physical, and color parameters of composite breads.

The purpose of the correlation analysis was to explore the relationships between instrumental, physicochemical, and compositional parameters (e.g., proximate composition, phenolic content, color parameters, elasticity, porosity, and phytic acid) in order to better understand how lentil-derived ingredients influence bread structure and quality from a technological and nutritional perspective (Figure 3). Pearson’s correlations were therefore used as a supportive analytical tool, aimed at identifying potential associations between measurable variables, rather than as a proxy for sensory perception.

The total phenolic content (TPC) exhibited strong positive correlations with protein, indicating that the incorporation of lentil seeds and microgreens substantially enhanced both the phenolic fraction and the nutritional density of the bread matrix. This association can be attributed to the localization of phenolic compounds within the protein–mineral complexes of legumes, particularly in whole seeds and germinated materials [34]. The observed trend confirms that enrichment with legume-based ingredients promotes the accumulation of nutritionally relevant bioactive compounds.

A significant negative correlation was observed between TPC and lipid content, suggesting that formulations richer in phenolic compounds tend to present lower lipid levels. This phenomenon may result from both a dilution effect caused by the increased contribution of non-lipid plant fractions and the potential interactions between phenolic compounds and lipids during dough processing and thermal treatment. Similar trends have been reported for functional bakery products enriched with legume flours and microgreens [26].

The phytic acid content showed a positive correlation with ash, reflecting its well-known mineral-chelating properties. Conversely, negative correlations were observed between phytic acid and TPC, lipids and proteins, particularly in samples containing germinated lentils. These results indicate the effectiveness of germination and sourdough fermentation in reducing phytate levels through endogenous and microbial phytase activity, while simultaneously increasing the availability of phenolic compounds [35].

The chromatic parameters a*, b*, and chroma (C) were positively correlated with TPC and protein content, suggesting that color intensification is closely linked to the accumulation of phenolic compounds and the formation of Maillard reaction products. In contrast, lightness (L) exhibited significant negative correlations with TPC, protein, and phytic acid, indicating progressive darkening of the crumb as the concentration of bioactive compounds increased.

The hue angle (h°) was negatively correlated with a*, b*, and C, confirming the shift from lighter yellowish tones to darker reddish-brown hues in breads enriched with lentil ingredients and subjected to sourdough fermentation. This behavior is consistent with previous reports on functional breads enriched with legumes and fermented using traditional biotechnological processes.

Elasticity (%) and porosity (%) were positively correlated with protein, TPC, and color indices but negatively correlated with ash, lipids, and phytic acid. Increasing the protein content, even when derived from non-gluten sources, can improve the water binding capacity and dough cohesion, indirectly supporting gas retention during fermentation and baking. This mechanism explains the positive association between protein levels and higher values of elasticity and porosity [36]. The positive correlation with TPC and color indices is closely linked to the addition of microgreens, which are an important source of phenolic compounds and pigments. These compounds can interact with the protein–starch matrix, influencing the rheological behavior of the dough, and color indices can be considered indirect markers of the degree of microgreen incorporation. Thus, samples with higher TPC values and more intense color also exhibited superior structural properties. In contrast, the negative correlations between elasticity and porosity and the content of minerals (ash), lipids, and phytic acid indicate that these components can negatively affect the formation of the dough’s structural network. A high ash content, associated with an increased level of minerals, can disrupt the continuity of the gluten network, and lipids can cover the surface of proteins, reducing the protein–protein interactions necessary for the development of elasticity [35].

Overall, the correlation patterns highlight that both the type of fermentation (yeast vs. sourdough) and the nature of the plant ingredient (seeds vs. microgreens) play a decisive role in shaping the nutritional profile and visual attributes of the final bread products. Sourdough fermentation favored phytate degradation and color development, while lentil microgreens contributed substantially to the enhancement of phenolic and protein contents. These interactions demonstrate the potential of combining germination and fermentation strategies to obtain bakery products with improved functional and sensory characteristics.

**Figure 4:**
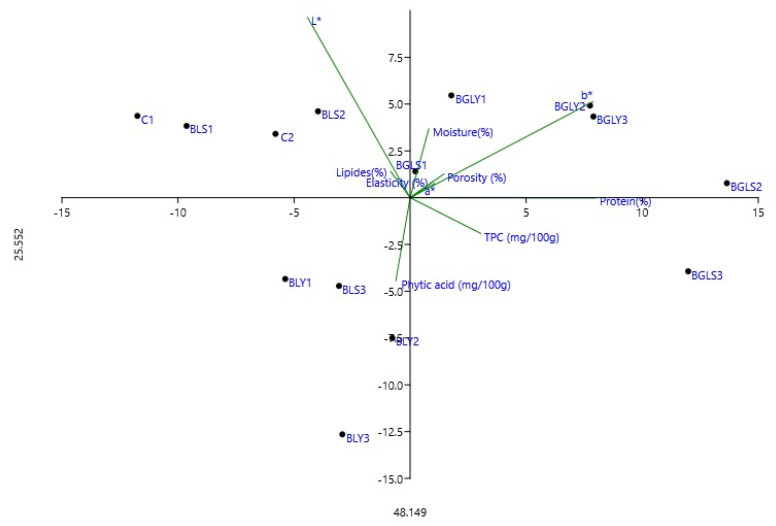
Principal Component Analysis (PCA) biplot illustrating the relationships between compositional, color, and phytochemical parameters of composite yeast- and sourdough-fermented breads enriched with lentil seeds and microgreens.

Principal Component Analysis (PCA) was applied to explore the multivariate relationships among the compositional and phytochemical parameters of composite breads obtained by yeast and sourdough fermentation with lentil seeds and microgreens addition. The PCA biplot (Figure 4) highlights the discrimination of samples according to the fermentation type and level of enrichment, as well as the contribution of total phenolic content (TPC), phytic acid, physical parameters and macronutrients to the sample variability.

The PCA results indicate that the first three principal components had eigenvalues higher than 1, fulfilling the Kaiser criterion and together explaining approximately 91.59% of the total data variability. Of these, the first two principal components were considered relevant for graphical interpretation, as they cumulatively explain 73.67% of the total variation (PC1 = 48.15%, eigenvalue = 60.24; PC2 = 25.52%, eigenvalue = 31.93), which is confirmed in a two-dimensional space.

Principal component 1 (PC1), which explains the largest proportion of variability, is predominantly associated with nutritional and structural parameters. High positive loadings were observed for protein content, porosity, total phenolic compound (TPC) content, and chromatic component b*, while negative loadings were associated with lipid content and product elasticity. This distribution suggests that PC1 describes a gradient of nutritional and functional improvement, in which samples positioned on the positive side of the axis are characterized by higher values of proteins and antioxidant compounds, along with a more aerated structure, while samples located in the negative area are correlated with a denser matrix and higher lipid content.

Principal component 2 (PC2) is mainly defined by parameters related to color, moisture content, and the presence of antinutritional compounds, showing positive loads for lightness (L*) and moisture content and negative loads for phytic acid content. This axis reflects the differences between samples in terms of optical characteristics and antinutritional compound levels, indicating that samples located in the negative area of PC2 are associated with higher phytic acid values, while samples located at the top of the graph have higher brightness and a higher degree of moisture content.

The distribution of samples in the PC1–PC2 plane shows a clear separation between the control samples and germ-enriched samples. The control samples are predominantly grouped in the negative area of PC1, characterized by lower protein and TPC values, while the germ-enriched samples are positioned in the positive quadrants, indicating a strong correlation with improved nutritional and functional parameters. The opposite orientation of the phytic acid vector to proteins and TPC confirms the beneficial effect of the germination process on reducing antinutritional compounds, thus contributing to increased nutrient bioavailability. Overall, the PCA results demonstrate that the addition of sprouts significantly influences the nutritional, antioxidant, and structural profile of the products, supporting the functional potential of the developed formulations.

Overall, the PCA results demonstrate that fermentation type and raw material (seeds vs. microgreens) are the main drivers of variability among samples. The strong association between microgreen lentil breads and TPC, along with their separation from phytic acid-rich samples, highlights the nutritional advantage of using microgreen ingredients, especially in sourdough-fermented systems.

## 3. Materials and Methods

### 3.1. Preparation of Powder Seeds and Germinated Lentil as Microgreen

Two lentil-derived ingredients were used in this study: (i) lentil seed flour obtained from raw seeds and (ii) germinated lentil powder obtained from aerial vegetative (8–10 cm) parts after germination and growth under light conditions.

**Figure 5:**
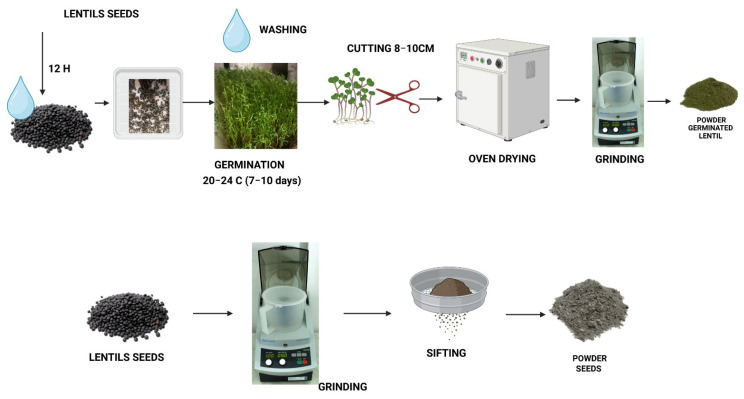
The technological flow used for sample preparation (Figure created with BioRender.com, accessed on 15 November 2025).

Lentil seeds were purchased from Pronat SRL in Timisoara (Romania) and were used to prepare lentil powders after grinding, sifting and mixing of lentil seeds, as is presented in Figure 5. The lentil microgreen powders were obtained from lentil seeds after germination, according to the procedure described in Figure 5.

### 3.2. Flours and Breads Preparation

Six types of flour composite were obtained: three based on wheat flour (WF) and lentil seed flour (LF): 10% lentil seeds + 90% wheat flour; 20% lentil seeds + 80% wheat flour; and 30% lentil seeds + 70% wheat flour and three based on wheat flour (WF) fortified with germinated lentil as microgreen powder (GLF) at 2.5, 5, and 7%. The selected substitution levels were based on the literature data and preliminary trials aimed at maintaining dough workability and bread quality [37,38,39,40,41,42]. The composite flours were obtained by mixing in different percentages of lentil seed powder and germinated lentil powder with wheat flour, as presented in Table 4.

**Figure 6:**
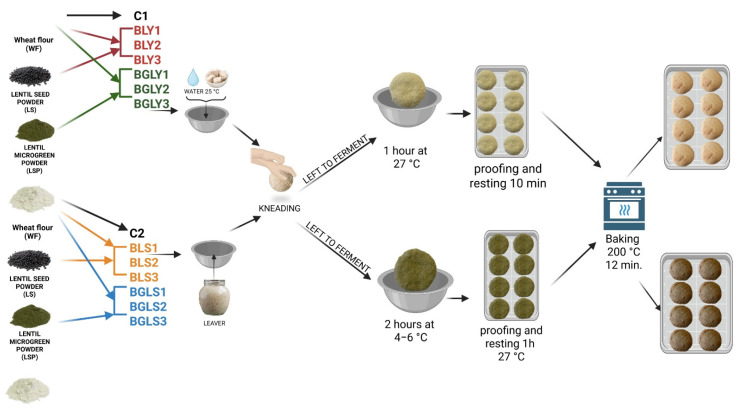
Technological process of obtaining composite breads (Figure created with BioRender.com, accessed on 15 November 2025).

The composite flours, consisting of wheat flour partially substituted with lentil seed or germinated lentil flours at different incorporation levels, were formulated prior to bread making. These composite flours were subsequently used to obtain bread by two different technological approaches: a direct bread-making method using baker’s yeast and an indirect method based on sourdough fermentation. The formulation of composite flours and the corresponding technological pathways are schematically illustrated in Figure 6. All ingredients used in the bread making (type 650 wheat flour, salt and active dry baker’s yeast) were sourced from the local supermarket Auchan in Timisoara, Romania.

All dry ingredients were first sieved to remove impurities and then gravimetrically dosed according to the experimental formulations (Table 5). The experimental process and the technological parameters are presented in Table 6.

In the case of sourdough bread samples, a wheat sourdough was prepared using wheat flour 650 and water. No lentil flour or sprouted lentil material was used during sourdough propagation in order to maintain a stable cereal-based microbial ecosystem. Lentil-derived ingredients were added only during final dough mixing. The sourdough microbiota consisted predominantly of lactic acid bacteria (LAB) belonging to the genera Lactobacillus spp., *Lactiplantibacillus,* and *Levilactobacillus*. Sourdough propagation was carried out through daily refreshments at a ratio of 1:1:1 (sourdough:flour:water, *w*/*w*/*w*) and fermented at 25 °C for 24 h.

At the time of use in bread making, the sourdough exhibited a pH of 4.3 and a total titratable acidity of 5.1 mL NaOH/10 g, indicating a mature and microbiologically stabilized fermentation state. Therefore, the sourdough was incorporated into the dough during the stabilized (stationary) phase of fermentation.

### 3.3. Evaluation of the Physical Properties of Bread Samples

The main physical parameters, such as the porosity, crumb elasticity, and height-to-diameter ratio (H/D) were evaluated for the bread samples, according to the SR 91:2007 standard [43]. All measurements were performed in triplicate for each sample.

The porosity was determined as the percentage of pore volume corresponding to 100 g of crumb and was calculated according to Equation (1):

(1) $$Porosity\left(\%,vol.\right)=\frac{V-\frac{m}{\rho }}{V}\;\times 100\;\;$$

where V represents the volume of the crumb cylinder (cm^3^), m is the mass of the crumb cylinder (g), and ρ is the density of the compact crumb (g/cm^3^). According to the SR 91:2007 standard [16], the value of *ρ* is 1.26 g/cm^3^ for semi-white wheat bread.

Crumb elasticity, as calculated using Equation (2), was assessed by compressing a cylindrical crumb sample (6 cm in height) to compression for 1 min, followed by measurement of its recovery.

(2) $$Elasticity\left(\%\right)=\frac{B}{A}\times 100$$

where *A* represents the height of the crumb cylinder before to compression, and *B* represents its height after it has recovered.

The height-to-diameter ratio (H/D) was assessed by measuring the maximum bread height (H, cm) and the average of two perpendicular diameters (D, cm) and was calculated using Equation (3):

(3) $$Height/Diameter\;ratio=\frac{H}{D}$$

### 3.4. Determination of Nutritional Composition

The methods used for nutritional parameters were protein, fat, total mineral substances and carbohydrates content, as presented in Table 7.

### 3.5. Determination of Antinutritional Compounds—Phytic Acid

Phytic acid was quantified using an enzymatic-colorimetric assay (K-PHYT Phytic Acid/Total Phosphorus Kit; Megazyme/Neogen, Lansing, MI, USA), following the manufacturer’s latest instructions (2025) [47].

For analysis, approximately 1.0 g of each sample was extracted with 0.66 M hydrochloric acid. After centrifugation, the extract was neutralized and treated sequentially with phytase to cleave phytic acid and with alkaline phosphatase to ensure complete release of inorganic phosphate. The amount of phosphate released was subsequently determined using the molybdenum blue reaction, with the absorbance being measured spectrophotometrically at 655 nm [48].

### 3.6. Determination of Total Polyphenol Content (TPC)

For the total polyphenol content (TPC), the Folin–Ciocalteu method [49] was used. For the extract, 1 g of product was weighed, and 10 mL of 70% aqueous ethanol was added and stirred for 30 min on a mechanical shaker (IDL, Freising, Germany). After the 30 min, 0.5 mL of extract was taken and mixed with Folin–Ciocalteu reagent (diluted 1:10) (Sigma-Aldrich Chemie GmbH, Munich, Germany) and 1 mL of sodium carbonate solution (60 g/L) (Geyer GmbH, Renningen, Germany).

After adding the reagents, it was incubated at 50 °C for 30 min using an INB500 incubator (Memmert GmbH, Schwabach, Germany). The absorbance was measured at 750 nm using a UV–Vis spectrophotometer, specifically the Specord 205 model (Analytik Jena AG, Jena, Germany). The results were expressed in mg GAE/100 g sample. Each sample was determined in triplicate.

### 3.7. Determination of Color Parameters

All color measurements were, therefore, performed on fresh bread samples (24 h after baking). The color characteristics of bread products were evaluated using a colorimeter (Konica Minolta, Tokyo, Japan), using the CIE L a b* color system, internationally recognized for colorimetric analysis. In this system, the L* coordinate indicates the brightness (0 = black, 100 = white), the a* parameter describes the chromatic variation on the green (−)–red (+) axis, and b* reflects the variation on the blue (−)–yellow (+) axis [50].

In addition to the fundamental coordinates L *, a *, and b *, derived chromatic parameters were calculated, namely chroma (C *), associated with the intensity or saturation of the color, and hue angle (h°), which expresses the visually perceived chromatic tone. For determinations, bread samples were portioned into sections, and measurements were performed at several points of each sample, to obtain representative average values. All analyses were performed in triplicate [36].

### 3.8. Statistical Analysis

The results are presented as mean values ± standard deviation (SD). Differences between means were assessed using Tukey HSD, JASP 0.95.4, followed by a multiple comparison analysis performed with a t-test in Microsoft Excel 365. Statistical significance was set at *p* < 0.05. All determinations were performed in triplicate.

## 4. Conclusions

This study provides a comprehensive evaluation of the combined effects of lentil germination and fermentation strategy on the nutritional, antinutritional, physical, and technological quality of composite wheat breads. The results clearly demonstrate that both the form of lentil incorporation (seeds vs. microgreens) and the fermentation regime (yeast vs. sourdough) act as decisive factors shaping the final bread characteristics.

Partial substitution of wheat flour with lentil ingredients led to a marked improvement in nutritional quality, particularly in terms of protein and mineral content. This confirms the high nutritional potential of lentil microgreens and highlights the synergistic role of sourdough fermentation in enhancing protein retention and concentration.

The applied bioprocessing strategies significantly affected the antinutritional factors. The phytic acid content was substantially lower in breads containing lentil microgreens than in those formulated with lentil seeds. Moreover, sourdough fermentation consistently reduced the phytic acid levels by approximately 15–25% compared to yeast fermentation across all formulations. These findings demonstrate the effectiveness of combining germination and sourdough fermentation as complementary approaches for limiting phytate accumulation and potentially improving the mineral bioavailability.

The total polyphenol content (TPC) increased significantly with lentil addition, with the highest values observed in sprout-enriched breads. Sourdough fermentation further enhanced TPC in sprout-based formulations, whereas seed-based sourdough breads exhibited lower TPC compared to their yeast-fermented counterparts. This matrix-dependent response highlights the critical role of germination in promoting phenolic release and stabilization during lactic fermentation.

The technological properties were also affected by the applied processing strategies. Breads fermented with sourdough showed higher moisture retention, improved porosity, elasticity, and height-to-diameter ratios compared to yeast-fermented samples. The most favorable physical characteristics were consistently observed in formulations with intermediate lentil incorporation levels (20% seeds or 7.5% microgreens), indicating an optimal balance between nutritional enhancement and structural performance.

Multivariate analysis supported these observations. Pearson correlations and PCA revealed a strong positive association between total polyphenol content, protein, and chromatic parameters, while phytic acid was inversely related to TPC and protein, particularly in sprout-based sourdough breads. PCA clearly discriminated samples according to fermentation type and lentil form, clustering sprout-enriched sourdough breads with high TPC and reduced phytic acid, thus confirming the nutritional advantage of this formulation strategy.

Overall, the results demonstrate that the combined use of lentil microgreens and sourdough fermentation represents the most effective approach for producing composite wheat breads with enhanced protein content, elevated polyphenol levels, reduced antinutritional factors, and improved technological quality. These findings provide a scientifically grounded basis for the development of cereal–legume bakery products with improved functional and nutritional value. Future research should focus on optimizing fermentation duration and starter composition and on evaluating mineral bioaccessibility and in vivo antioxidant effects to further substantiate the health potential of these breads. Studies on sensory analysis and consumer perception regarding bakery products with sprouted and unsprouted lentil additions are also of future interest in the context of technology transfer and product commercialization.

## Figures and Tables

**Table 1:** Physical characteristics of bread obtained with WF fortified with LF and GLF using yeast and sourdough fermentation.

Bread Samples	Elasticity (%)	Porosity (%)	Height/Diameter (H/D)
Bread fermented with yeast			
C1	91.220 ± 0.200 ^a^	66.087 ± 0.153 ^a^	0.526 ± 0.011 ^a^
BLY1	91.857 ± 0.153 ^a^	67.790 ± 0.173 ^a^	0.555 ± 0.009 ^a^
BLY2	92.267 ± 0.050 ^a^	68.100 ± 0.106 ^a^	0.569 ± 0.005 ^a^
BLY3	91.440 ± 0.115 ^a^	66.177 ± 0.055 ^a^	0.534 ± 0.014 ^a^
BGLY1	93.073 ± 0.058 ^a^	68.340 ± 0.265 ^a^	0.578 ± 0.012 ^a^
BGLY2	94.153 ± 0.153 ^a^	69.680 ± 0.178 ^a^	0.588 ± 0.004 ^a^
BGLY3	91.625 ± 0.132 ^a^	66.610 ± 0.204 ^a^	0.577 ± 0.011 ^a^
Bread fermented with sourdough			
C2	92.140 ± 0.132 ^a^	67.607 ± 0.202 ^a^	0.546 ± 0.007 ^a^
BLS1	92.827 ± 0.101 ^a^	68.640 ± 0.150 ^a^	0.570 ± 0.010 ^a^
BLS2	93.647 ± 0.252 ^a^	71.173 ± 0.153 ^b^	0.586 ± 0.008 ^a^
BLS3	92.493 ± 0.050 ^a^	67.220 ± 0.100 ^a^	0.558 ± 0.011 ^a^
BGLS1	93.727 ± 0.095 ^a^	69.760 ± 0.115 ^a^	0.588 ± 0.004 ^a^
BGLS2	94.920 ± 0.075 ^a^	72.607 ± 0.202 ^b^	0.590 ± 0.007 ^a^
BGLS3	92.763 ± 0.104 ^a^	68.763 ± 0.056 ^a^	0.574 ± 0.020 ^a^

The values are expressed as mean ± standard deviation; data within the same column sharing different superscripts are significantly different (*p* < 0.05).

**Table 2:** The nutritional parameters of bread obtained with WF fortified with LF and GLF using yeast and sourdough fermentation.

Samples				Nutritional Characteristics		
	Moisture	Ash	Protein	Fat	Carbohydrates	Energy Value (kcal/100 g)
	(%)	(%)	(%)	(%)	(g/100 g)	
Composite bread with yeast fermentation						
C1	35.01 ± 0.68 ^a^	0.89 ± 0.02 ^a^	12.71 ± 0.99 ^a^	2.61 ± 0.03 ^a^	48.77 ± 0.29 ^a^	269.47 ± 2.77 ^a^
BLY1	32.69 ± 0.45 ^b^	1.18 ± 0.08 ^b^	17.40 ± 2.34 ^b^	1.07 ± 0.03 ^b^	47.66 ± 2.78 ^a^	269.87 ± 1.97 ^a^
BLY2	29.45 ± 0.23 ^c^	1.52 ± 0.12 ^b^	18.16 ± 3.00 ^b^	0.75 ± 0.53 ^b^	50.12 ± 2.82 ^a^	279.87 ± 1.92 ^b^
BLY3	26.28 ± 0.11 ^b,c^	2.23 ± 0.28 ^c^	19.72 ± 0.11 ^b^	0.45 ± 0.10 ^b^	51.32 ± 0.40 ^a^	288.21 ± 1.95 ^b^
BGLY1	33.53 ± 0.60 ^b^	0.69 ± 0.42 ^a^	21.33 ± 0.51 ^c^	1.22 ± 0.06 ^b^	43.23 ± 1.12 ^a^	269.22 ± 3.06 ^a^
BGLY2	32.80 ± 5.59 ^b^	1.23 ± 0.20 ^b^	24.44 ± 0.41 ^c^	1.82 ± 0.09 ^c^	39.71 ± 5.77 ^a^	272.98 ± 22.68 ^b^
BGLY3	31.64 ± 0.32 ^b^	1.99 ± 0.21 ^c^	26.53 ± 0.87 ^c^	1.93 ± 0.04 ^c^	37.91 ± 0.80 ^a^	275.13 ± 0.58 ^b^
Composite bread with sourdough fermentation						
C2	29.31 ± 0.56 ^a^	1.13 ± 0.06 ^a^	16.81 ± 2.95 ^a^	2.85 ± 0.05 ^a^	49.89 ± 2.93 ^a^	292.52 ± 2.65 ^a^
BLS1	39.70 ± 0.29 ^b^	1.46 ± 0.46 ^b^	14.50 ± 0.13 ^a^	3.91 ± 0.07 ^b^	40.42 ± 0.78 ^b^	254.90 ± 2.61 ^b^
BLS2	41.72 ± 0.46 ^c^	1.47 ± 0.25 ^b^	18.23 ± 0.29 ^b^	2.08 ± 0.03 ^a^	36.50 ± 0.47 ^c^	237.71 ± 2.52 ^c^
BLS3	41.03 ± 1.03 ^c^	2.67 ± 0.25 ^c^	20.57 ± 0.05 ^c^	1.07 ± 0.97 ^c^	34.66 ± 2.10 ^c^	230.57 ± 3.16 ^c^
BGLS1	35.72 ± 0.63 ^bc^	1.14 ± 0.07 ^a^	23.00 ± 0.40 ^c^	0.56 ± 0.06 ^c^	47.96 ± 13.51 ^a^	288.93 ± 55.02 ^a^
BGLS2	38.23 ± 0.29 ^b^	1.15 ± 0.12 ^a^	26.81 ± 2.67 ^d^	0.89 ± 0.02 ^c^	33.22 ± 2.92 ^c^	248.13 ± 1.30 ^c^
BGLS3	38.53 ± 0.13 ^b^	1.19 ± 0.18 ^a^	29.18 ± 0.50 ^d^	1.03 ± 0.07 ^c^	30.37 ± 0.44 ^c^	247.47 ± 1.67 ^c^

The values are expressed as mean ± standard deviation; data within the same column, in the same group (bread with yeast fermentation or bread with sourdough fermentation), sharing different superscripts, are significantly different (*p* < 0.05).

**Table 3:** Color parameters of bread obtained with WF fortified with LF and GLF using yeast and sourdough fermentation.

Samples	L*	a*	b*	C*	h°
Composite bread with yeast fermentation					
C1	64.58 ± 0.21 ^a^	0.50 ± 0.02 ^a^	13.25 ± 0.78 ^a^	13.26 ± 0.13 ^a^	87.84 ± 0.10 ^a^
BLY1	56.09 ± 0.45 ^a^	0.31 ± 0.04 ^a^	12.21 ± 0.41 ^a^	12.21 ± 0.04 ^a^	88.55 ± 0.14 ^a^
BLY2	52.89 ± 1.12 ^a^	1.07 ± 0.01 ^a^	17.70 ± 1.12 ^a^	17.73 ±0.21 ^a^	86.54 ± 0.20 ^a^
BLY3	50.13 ± 1.41 ^a^	0.54 ± 0.04 ^a^	11.29 ± 1.47 ^a^	11.30 ±0.07 ^a^	87.26 ± 0.13 ^a^
BGLY1	61.96 ± 0.47 ^a^	1.77 ± 0.14 ^a^	22.77 ± 1.96 ^a^	22.84 ± 0.09 ^a^	85.56 ± 0.20 ^a^
BGLY2	60.18 ± 0.05 ^a^	3.16 ± 0.69 ^a^	24.77 ± 1.65 ^a^	24.97 ± 0.15 ^a^	82.73 ± 0.08 ^a^
BGLY3	60.07 ± 0.31 ^a^	2.74 ± 0.12 ^a^	24.95 ± 1.34 ^a^	25.10 ± 0.23 ^a^	83.73 ± 0.10 ^a^
Mean	57.99	1.44	18.13	18.20	86.03
SD	5.16	1.14	6.03	6.10	2.15
Composite bread with sourdough fermentation					
C2	62.32 ± 0.10 ^a^	1.22 ± 0.7 ^a^	17.81 ± 0.12 ^a^	17.85 ± 0.10 ^a^	86.08 ± 0.45 ^a^
BLS1	62.70 ± 1.02 ^a^	0.17 ± 0.9 ^b^	12.22 ± 0.98 ^a^	12.22 ± 0.35 ^a^	89.20 ± 0.12 ^a^
BLS2	62.35 ± 1.32 ^a^	1.04 ± 0.10 ^a^	15.76 ± 0.65 ^a^	15.79 ± 1.25 ^a^	86.22 ± 1.98 ^a^
BLS3	54.89 ± 0.14 ^b^	0.67 ± 1.54 ^a^	11.46 ± 0.10 ^a^	11.48 ± 2.14 ^a^	86.65 ± 2.79 ^a^
BGLS1	59.67 ± 0.21 ^c^	0.37 ± 2.10 ^b^	15.91 ± 0.96 ^a^	15.91 ± 1.98 ^a^	88.67 ± 1.22 ^a^
BGLS2	51.34 ± 0.28 ^d^	1.53 ± 1.87 ^a^	27.71 ± 0.10 ^b^	27.75 ± 2.73 ^b^	86.84 ± 1.63 ^a^
BGLS3	49.30 ± 0.05 ^d^	1.66 ± 1.65 ^a^	21.90 ± 0.32 ^b^	21.96 ± 1.32 ^b^	85.67 ± 2.36 ^a^
Mean	57.51	0.95	17.54	17.57	87.05
SD	5.63	0.57	5.68	5.70	1.35

Values are expressed as mean ± SD. Means in the same column and the same group (bread with yeast fermentation or bread with sourdough fermentation) followed by the same letter are not significantly different according to Tukey’s HSD test (*p* > 0.05).

**Table 4:** The flours and bread composition and the sample codes.

Bread Sample	Flour	Cod Sample
Yeast control bread	100% wheat flour	C1
Sourdough control bread	100% wheat flour	C2
Yeast lentil seeds bread 10%	10% lentils seeds + 90% wheat flour	BLY1
Yeast lentil seeds bread 20%	20% lentils seeds + 80% wheat flour	BLY2
Yeast lentil seeds bread 30%	30% lentils seeds + 70% wheat flour	BLY3
Sourdough lentil seeds bread 10%	10% lentils seeds + 90% wheat flour	BLS1
Sourdough lentil seeds bread 20%	20% lentils seeds + 80% wheat flour	BLS2
Sourdough lentil seeds bread 30%	30% lentils seeds + 70% wheat flour	BLS3
Yeast germinated lentil bread 2.5%	2.5% germinated lentils + 97.5% wheat flour	BGLY1
Yeast germinated lentil bread 5%	5% germinated lentils + 95% wheat flour	BGLY2
Yeast germinated lentil bread 7.5%	7.5% l germinated lentils + 92.5% wheat flour	BGLY3
Sourdough germinated lentil bread 2.5%	2.5% germinated lentils + 97.5% wheat flour	BGLS1
Sourdough germinated lentil bread 5%	5% germinated lentils + 95% wheat flour	BGLS2
Sourdough germinated lentil bread 7.5%	7.5% germinated lentils + 92.5% wheat flour	BGLS3

**Table 5:** The recipes for bread preparation.

Ingredients	Wheat Flour (kg)	Lentil Flour (kg)	Germinated Lentil Powder (kg)	Active Dry Baker’s Yeast (kg)	Sourdough (kg)	Salt (Kg)	Water (mL)
C1	1	-	-	0.030	-	0.020	500
BLY1	0.900	0.100	-	0.030	-	0.020	500
BLY2	0.800	0.200	-	0.030	-	0.020	500
BLY3	0.700	0.300	-	0.030	-	0.020	500
BGLY1	0.975	-	0.025	0.030	-	0.020	500
BGLY2	0.950	-	0.050	0.030	-	0.020	500
BGLY3	0.925	-	0.075	0.030	-	0.020	500
C2	1	-	-	-	0.200	0.020	500
BLS1	0.900	0.100	-	-	0.200	0.020	500
BLS2	0.800	0.200	-	-	0.200	0.020	500
BLS3	0.700	0.300	-	-	0.200	0.020	500
BGLS1	0.975	-	0.025	-	0.200	0.020	500
BGLS2	0.950	-	0.050	-	0.200	0.020	500
BGLS3	0.925	-	0.075	-	0.200	0.020	500

**Table 6:** Technological process and parameters of baking.

Steps	Process	Parameters	
Yeast fermentation	Active dry baker’s yeast (*Saccharomyces cerevisiae*) was dissolved in water prior to mixing.	Yeast pH: 4.25; Water Temperature: 25 °C Time: 30 min	
Sourdough fermentation	Sourdough-fermented samples were prepared using an active natural sourdough starter containing lactic acid bacteria (mainly *Lactobacillus* spp., *Lactiplantibacillus*, *Levilac-tobacillus*).	Sourdough pH: 4.3 Sourdough Total Titratable Acidity (TTA): 5.1 mL NaOH/10 g Sourdough Temperature: 25 °C Time: 24 h	
Mixing	Dough mixing was carried out in two stages: initial mixing to ensure homogenization, followed by intensive mixing to promote gluten development. Salt was added during the second mixing stage.	1st stage	2nd Stage
		Speed: 80 rpm Time: 4 min	Speed: 160 rpm Time: 4 min
Dough fermentation with yeast	Yeast-fermented doughs were processed according to a conventional fermentation procedure (direct method without sourdough).	Time: 1 h Temperature: 27 °C Humidity: 70%	
Dough fermentation with sourdough	Sourdough doughs underwent a prolonged fermentation step to enhance microbial activity and biochemical transformations.	Time:2 h Temperature: 4–5 °C Humidity: 70%	
Proofing	After fermentation, the doughs were kneaded, divided, scaled, and manually molded. Final proofing was performed at different parameters for yeast and sourdough.	Yeast Samples	Sourdough Samples
		Time: 1 h Temperature: 27 °C Humidity: 65%	Time: 10 min Temperature: 27 °C humidity: 70%
Baking	Baking was carried out in bakery oven, followed by cooling at room temperature prior to analysis.	Temperature: 200 °C Time: 12 min	

**Table 7:** The methods used for nutritional composition of flours and breads.

Parameters	Methods	Reference
Moisture	ICC Standard Methods (2003)	[44]
Protein content * (%)	ICC Standard Methods (2003)	[44]
Ash content * (%)	ISO Method No. 2171:2007	[45]
Fat content * (%)	AOAC Official Method (2000)	[46]
Carbohydrate content (g/100 g)	Calculated by difference: 100 − (moisture + ash + proteins + fats)	[37]
Energy value (kcal/100 g)	Calculated using the formula: Energy value = 4 × proteins + 4 × carbohydrates + 9 × lipids	[41]

* Calculated based on dry matter.

## Data Availability

The original contributions presented in the study are included in the article; further inquiries can be directed to the corresponding authors.
